# Higher Wheal Sizes of *Dermatophagoides farinae* Sensitization Exhibit Worse Nasal Symptoms in Allergic Rhinitis Patients

**DOI:** 10.3389/fmed.2022.843432

**Published:** 2022-02-28

**Authors:** Siti Muhamad Nur Husna, Norasnieda Md Shukri, Hern-Tze Tina Tan, Noor Suryani Mohd Ashari, Kah Keng Wong

**Affiliations:** ^1^Department of Immunology, School of Medical Sciences, Universiti Sains Malaysia, Kubang Kerian, Malaysia; ^2^Department of Otorhinolaryngology, Head and Neck Surgery, School of Medical Sciences, Universiti Sains Malaysia, Kubang Kerian, Malaysia; ^3^Hospital Universiti Sains Malaysia, Kubang Kerian, Malaysia

**Keywords:** allergic rhinitis, wheal size, *Dermatophagoides farinae*, nasal symptoms, house dust mite

## Abstract

Allergic rhinitis (AR) is a global health burden and it manifests in both nasal and non-nasal symptoms. Skin prick test (SPT) is a routine procedure to diagnose AR sensitized to common allergens including house dust mites (HDMs). The degree of sensitivity of a patient toward allergens is determined by the size of the wheal formed by SPT procedure. SPT wheal sizes are influenced by recent anti-histamine usage, however it remains unclear if SPT wheal sizes are also influenced by other factors. In this study, we set out to investigate the association between SPT wheal sizes with the demographical, clinical and environmental characteristics, as well as nasal and non-nasal symptoms severity scores, of AR patients (*n* = 30) sensitized to common HDMs (i.e., *Dermatophagoides pteronyssinus, Dermatophagoides farinae*, and *Blomia tropicalis*). We showed that SPT wheal sizes of HDM allergens were not associated with clinical, demographical and environmental characteristics examined. Nonetheless, significant correlations were observed between SPT wheal sizes of *D. farinae* sensitization with worse severity scores of all five nasal symptoms examined (i.e., sneezing, runny nose, itchy nose, congestion and postnasal drip) and four of the six non-nasal symptoms examined (i.e., throat symptoms, ear symptoms, headache and mental function). Such relationships were not observed in SPT wheal sizes of *D. pteronyssinus* and *B. tropicalis* sensitization. We suggest that increased SPT wheal sizes for *D. farinae* sensitization may predict the likelihood of more severe nasal and, to a lesser extent, non-nasal manifestations in AR patients.

## Introduction

Allergic rhinitis (AR) is a global health burden that affects ~400 million people globally (1, 2). The disease significantly derails the quality of life and requires persistent treatment, leading to social-economic consequences. Moreover, AR is frequently accompanied with comorbidities such as asthma, sinusitis and conjunctivitis, complicating treatment and management of AR patients (3, 4).

Both skin prick test (SPT) and specific immunoglobulin E (sIgE) immunoassay have good correlation in terms of specificity and sensitivity in the diagnosis of sensitization to common allergens (5–7). The main house dust mite (HDM) allergens AR patients sensitized to include *Dermatophagoides pteronyssinus* (*D. pteronyssinus*)*, Dermatophagoides farinae* (*D. farinae*) and *Blomia tropicalis* (*B. tropicalis*), and ~80% of AR patients are sensitized to these three HDM species (7–11).

The general principles of SPT include the introduction of relevant allergens into the skin, causing sIgE to bind surface receptors on mast cells, inducing mast cells to degranulate and release of inflammatory mediators including histamine (12). Histamine is responsible to promote capillary dilation, increases vascular permeability, induces pain nociceptors, and stimulates eosinophil chemotaxis to the inflamed tissue. The exudation then enters the skin and causes swelling accompanied by itching (12, 13). As a result, this produces a wheal and flare response whose diameter can be measured.

Allergen sensitization is a key risk factor for the development of allergic diseases. Therefore, it is essential to determine the individual's allergen sensitization for the diagnosis and treatment of allergies (14). Characteristics such as environmental factors (e.g., geographical area) and demographic factors (e.g., age and sex) influence the pattern of inhalant allergen (including HDM) sensitization (15–19). The degree of sensitivity of a patient toward allergens is ascertained by the size of the wheal formed during SPT (20).

Nevertheless, it remains unclear if SPT wheal sizes could predict symptoms severity of AR patients (21, 22) or if they are influenced by factors other than recent anti-histamine usage or treatments. These factors include demographical, clinical and environmental characteristics, as well as nasal and non-nasal symptoms severity scores, of AR patients sensitized to HDMs. These intrinsic and extrinsic characteristics may influence or reflect SPT wheal sizes. Thus, this study attempted to examine these characteristics that might be associated with increased degree of sensitivity reflected by SPT wheal sizes in HDM-sensitized AR patients.

## Materials and Methods

### Study Population

The study population was part of our previously published cross-sectional gene expression study of AR patients (*n* = 30) conducted from February 2019 until January 2020 at the Otorhinolaryngology, Head and Neck Surgery (ORL-HNS) clinic in Hospital Universiti Sains Malaysia (HUSM), and research laboratory of Department of Immunology, Universiti Sains Malaysia (USM) (23). Diagnosis of AR was conducted using SPT against the allergens from three HDM species (i.e., *D. pteronyssinus, D. farinae* and *B. tropicalis*). The mean age of the AR cohort was 28.7 years old consisting of 10 males and 20 females, and the complete clinico-demographical and environmental characteristics have previously been published (23). All subjects were recruited with signed informed consent and the study protocols were approved by the Human Research Ethics Committee of Universiti Sains Malaysia (JEPeM) (approved code: USM/JEPeM/18060273).

### Skin Prick Test

Our exclusion criteria mandated our study subjects to stop using anti-allergy agents including steroids, anti-histamine and leukotriene receptor antagonists in the recent two weeks before the sample collection procedures were conducted. The study participants were screened using SPT to examine their sensitization toward HDM allergens (*D. farinae, D. pteronyssinus* or *B. tropicalis*). The forearm of study's subject was examined for any non-specific rash (i.e., without allergic/atopic characteristics) and chronic urticaria in the absence of allergic features on history. The study's subject had no food intolerance without allergic features (e.g., irritable bowel syndrome), chronic fatigue without allergic features, migraine headaches, behavioral disorders and reactions to respiratory irritants (e.g., smoke, fumes, perfumes). The study's subject was also asked about their medication status as it may interfere with SPT responses and the drugs may be contraindicated in skin testing.

Firstly, the test was initiated by sterilizing the prick area beforehand using an alcohol swab (i.e., not essential in the case of extreme dry skin and eczema). The forearm of the participant was pricked using intradermal sterile lancet in five separate pricks. The forearm was labeled with five different sections, positive control (+), negative control (–), *D. farinae* (f), *D. pteronyssinus* (p) and *B. tropicalis* (b) by using a skin marker pen. One drop of each allergen tested, histamine as positive control and saline as negative control was placed at the pricked area. The pricked area was observed for about 15–30 min to detect the sensitization toward the allergens. The size of wheals was measured using a ruler and was recorded. The patients with wheal size of 4 mm and more were considered positive for sensitization. A total of 30 AR patients met these criteria and were recruited in this study.

### Nasal and Non-nasal Symptoms Severity Scores Assessment

The patients were assessed with AR nasal and non-nasal symptoms severity scores. The nasal symptoms assessed were sneezing, runny nose, itchy nose, congestion (stuffiness) and postnasal drip. Non-nasal symptoms assessed were throat symptoms, chronic cough, eye symptoms, ear symptoms, headache and mental function. The 7-point visual analog scale (VAS) was used in these assessments (24) (Supplementary Table 1).

### Statistical Analysis

The numerical data for the clinico-demographical and environmental characteristics of AR patients and healthy controls were described using median and interquartile rage (IQR). Correlation analysis between two continuous variables was conducted using the Spearman correlation coefficient. For comparison of each characteristic within the three groups (i.e., wheal sizes according to the sensitization to each of the three HDMs), Dunn's multiple comparisons *post hoc* test was performed following Kruskal-Wallis test. All statistical analysis was conducted using GraphPad Prism (v6; GraphPad Software Inc., USA). For all tests, two-tailed *p* < 0.05 was considered to be statistically significant.

## Results

### Association of SPT Wheal Sizes of HDM Allergens With Demographical, Clinical and Environmental Characteristics of AR Patients

The associations of wheal sizes of HDM allergens (*D. pteronyssinus, D. farinae* and *B. tropicalis*) with demographical (age, sex), clinical (family history of allergic diseases, classification of AR, comorbidities) and environmental (exposure to secondhand smoke, home location, having pets, frequency of housekeeping, frequency of changing bedsheet) characteristics of AR patients were investigated. No significant difference was observed in all associations examined (Table 1).

**Table 1:** Association of clinico-demographical and environmental characteristics with SPT wheal sizes of HDM allergens in AR patients (*n* = 30).

**Characteristics**		**Wheal size (** ***D. pteronyssinus*** **)**		**Wheal size (** ***D. farinae*** **)**		**Wheal size (** ***B. tropicalis*** **)**	
		***r*-value or median (IQR)**	***p*-value**	***r*-value or median (IQR)**	***p*-value**	***r*-value or median (IQR)**	***p*-value**
Age (median: 27.5 years; IQR: 22.0–35.0)		*r* = 0.174	0.532	*r* = 0.340	0.215	*r* = 0.027	0.923
BMI (median: 26.5; IQR:23.3–28.6)		*r* = −0.488	0.065	*r* = −0.127	0.652	*r* = 0.308	0.264
Gender	Male	5.0 (2.0–11.0)	0.054	6.0 (3.0–10.3)	0.721	5.0 (2.3–11.0)	0.671
	Female	4.0 (2.3–5.0)		5.0 (4.0–9.0)		5.0 (3.0–6.8)	
Family history of allergic disease	Yes	4.0 (3.0–5.0)	0.481	5.0 (4.0–10.0)	0.523	5.0 (3.0–7.5)	0.548
	No	3.0 (0.0–9.0)		3.0 (1.5–10.0)		4.0 (1.5–8.0)	
Classification of AR	Persistent	4.0 (2.0–6.0)	>0.999	7.0 (3.5–10.0)	0.609	5.0 (4.0–9.0)	0.058
	Intermittent	4.0 (3.5–5.0)		5.0 (4.0–8.5)		3.0 (0.0–6.5)	
Comorbidity (pharyngitis)	Yes	4.0 (2.3–5.0)	0.597	7.0 (4.0–10.0)	0.627	5.0 (3.0–7.8)	0.831
	No	4.5 (2.8–6.5)		5.0 (3.8–10.0)		5.0 (2.3–7.3)	
Comorbidity (asthma)	Yes	5.0 (2.0–8.0)	0.214	7.0 (4.0–10.0)	0.316	5.0 (4.0–11.0)	0.214
	No	4.0 (3.0–5.0)		5.0 (3.0–10.0)		5.0 (3.0–7.0)	
Comorbidity (sinusitis)	Yes	4.0 (2.23–5.8)	0.871	5.0 (4.0–10.0)	0.641	5.0 (3.0–7.8)	0.366
	No	4.5 (4.0–5.0)		7.5 (4.0–11.0)		2.5 (0.0–5.0)	
Comorbidity (conjunctivitis)	Yes	4.0 (1.5–5.0)	0.062	5.0 (3.8–10.0)	0.457	5.0 (3.0–7.3)	0.567
	No	5.0 (4.3–7.5)		6.0 (4.0–10.8)		5.5 (1.0–12.3)	
Comorbidity (otitis media)	Yes	4.0 (1.0–4.5)	0.277	4.0 (0.0–10.0)	0.374	5.0 (3.5–6.0)	0.634
	No	5.0 (3.0–6.0)		5.0 (4.0–10.0)		5.0 (3.0–9.0)	
Exposure to secondhand smoke	Yes	4.0 (2.5–5.0)	0.662	5.0 (4.0–10.0)	0.681	5.0 (3.0–6.5)	0.125
	No	5.0 (1.5–7.0)		5.0 (2.0–9.0)		6.0 (4.0–12.0)	
Home location	Urban	4.0 (3.0–5.8)	0.893	6.0 (4.0–10.8)	0.295	5.0 (3.3–10.0)	0.582
	Rural	4.5 (2.0–5.3)		5.0 (3.8–8.0)		4.5 (3.0–7.3)	
Having pets	Yes	5.0 (1.5–6.0)	0.908	7.0 (4.5–10.5)	0.106	5.0 (3.0–9.5)	0.487
	No	4.0 (2.5–5.0)		4.0 (3.5–7.5)		4.0 (3.0–6.0)	
Frequency of housekeeping^*^	Daily	4.0 (0.0–5.0)	0.413	5.0 (4.0–8.0)	0.589	5.0 (3.0–8.0)	0.683
	Alternate daily	5.0 (2.0–5.0)		7.0 (3.0–10.0)		5.0 (4.0–5.0)	
	Weekly	5.0 (3.0–7.5)		6.0 (4.3–10.8)		6.0 (1.0 −11.0)	
Frequency of changing bedsheet^*^	Weekly	3.5 (0.5–4.8)	0.051	5.0 (3.3–6.5)	0.092	4.5 (3.0–9.0)	0.770
	Monthly	5.0 (3.8–6.8)		10.0 (4.0–10.3)		5.5 (3.0–7.3)	
	2-monthly	3.5 (0.8–4.8)		6.0 (1.0–11.0)		5.0 (4.3–9.5)	

* *The p-values were conducted according to Kruskal-Wallis test and all comparison between groups were insignificant by Dunn's multiple comparisons post hoc test*.

### Association of Nasal and Non-nasal Symptoms Severity Scores With the Number of HDM Allergens Sensitization of AR Patients

Any one of the three HDM allergens sensitized (mono-sensitized), two HDM allergens sensitized (double-sensitized), and three HDM allergens sensitized (triple-sensitized) represent the number of HDM allergens sensitization in these patients. No significant difference was observed for all associations investigated of nasal and non-nasal symptoms severity scores with the number of HDM allergens sensitization in AR patients. The association of nasal symptoms severity scores with the number of HDM allergens sensitization of AR patients are shown in Supplementary Figure 1. The association of non-nasal symptoms severity scores with the number of HDM allergens sensitization of AR patients are shown in Supplementary Figure 2.

### Correlation of Nasal and Non-nasal Symptom Severity Scores With SPT Wheal Sizes of HDM Allergens of AR Patients

**Figure 1:**
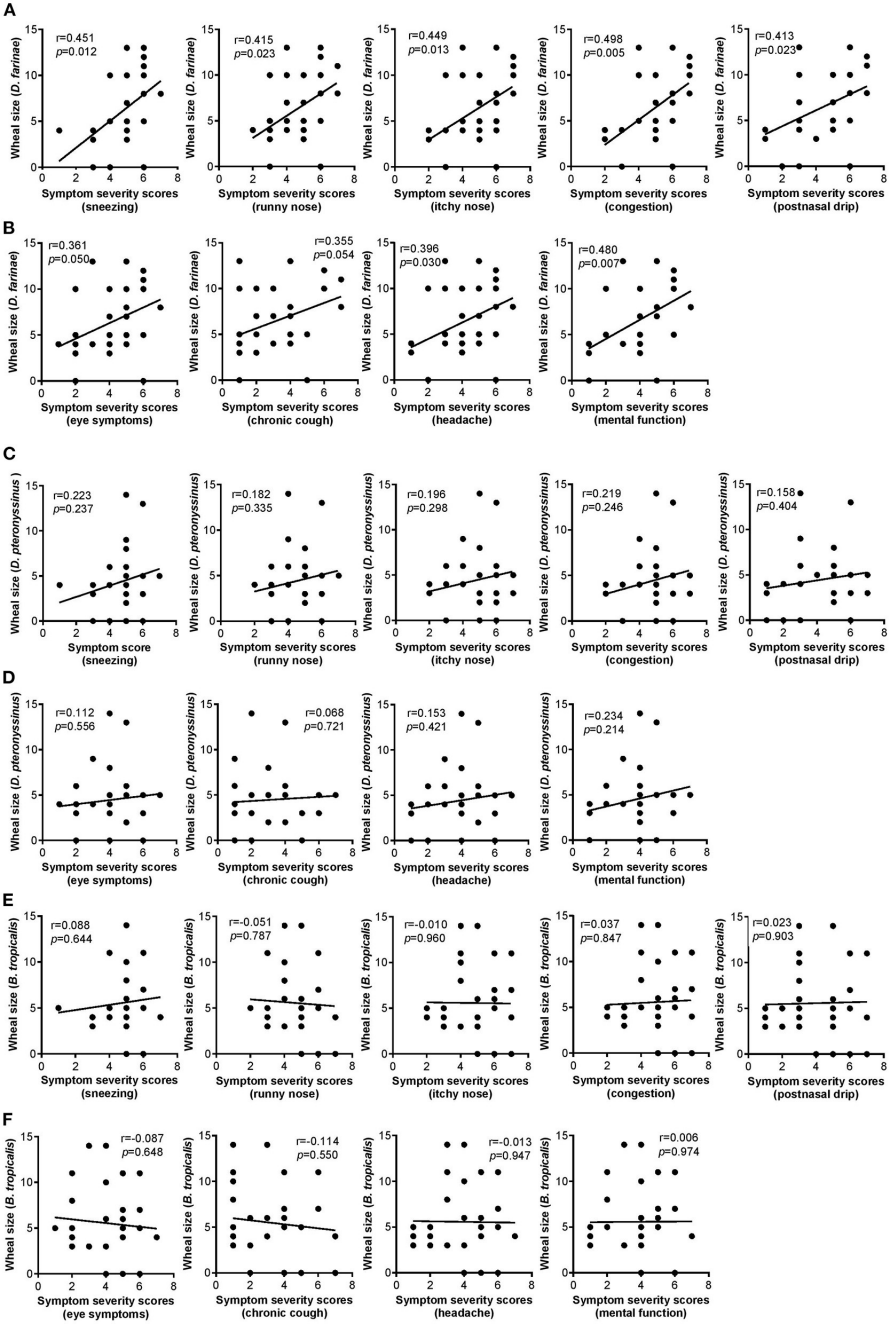
**(A,B)** Correlation of wheal sizes of *D. farinae* sensitization with nasal **(A)** and non-nasal **(B)** symptom severity scores in AR patients (*n* = 30). **(C,D)** Correlation of wheal sizes of *D. pteronyssinus* sensitization with nasal **(C)** and non-nasal **(D)** symptom severity scores in AR patients (*n* = 30). **(E,F)** Correlation of wheal sizes of *B. tropicalis* sensitization with nasal **(E)** and non-nasal **(F)** symptom severity scores in AR patients (*n* = 30).

The severity scores of all five nasal symptoms examined (i.e., sneezing, runny nose, itchy nose, congestion and postnasal drip) were positively and significantly correlated with the wheal sizes of AR patients sensitized to *D. farinae* (Figure 1A). Likewise, significant positive relationships were also observed between four of the six non-nasal symptoms examined (i.e., throat symptoms, ear symptoms, headache and mental function) and wheal sizes of AR patients sensitized to *D. farinae* (Figure 1B), except eye symptoms and chronic cough (data not shown for these insignificant correlations). Such significant relationships were not observed in the correlation of all the nasal and non-nasal symptoms' severity scores with wheal sizes of AR patients sensitized to *D. pteronyssinus* (Figures 1C,D) and *B. tropicalis* (Figures 1E,F).

## Discussion

In this study, no significant associations were observed between demographical, clinical and environmental characteristics with SPT wheal size in AR patients. We hypothesized that factors other than these characteristics such as nasal and non-nasal symptoms severity may be associated with SPT wheal sizes in each (or in combination) of the three HDMs sensitized. However, AR patients mono-sensitized, double-sensitized and triple-sensitized to HDM allergens did not show any significant difference in all nasal and non-nasal symptoms severity scores.

Polysensitization (i.e., sensitization to two or more allergens as confirmed by SPT) is associated with severe symptoms and poor QOL due to different immunologic phenotypes in monosensitized and polysensitized patients (25). In this study, symptoms severity scores were not associated with increased number of HDM sensitization. Polysensitized patients do not necessarily have polyallergy (i.e., documented, causal relationship between exposure to two or more specific, sensitizing allergens and the subsequent occurrence of relevant clinical symptoms of allergy), whereas all polyallergic patients are polysensitized (26). Our AR patients might not be polyallergic which may explain that the increased number of HDMs sensitization was not associated with increased symptoms severity scores.

Interestingly, almost all correlation of SPT wheal sizes of AR patients sensitized to *D. farinae* with nasal and non-nasal symptom severity scores showed significant positive correlations. These significant correlations were not observed in the rest of the HDM species examined (i.e., *D. pteronyssinus* and *B. tropicalis*). These results are comparable with previous report of asthma severity was significantly correlated with skin index of reactivity to *D. farinae* sensitization (27). Our observations of non-significance between *D. pteronyssinus* with any nasal symptoms also tally with an independent study demonstrating that SPT wheal sizes of AR patients sensitized to *D. pteronyssinus* did not correlate with the severity of nasal symptoms (28). Nonetheless, not all non-nasal symptoms (i.e., eye symptoms and chronic cough) were correlated with SPT wheal sizes for *D. farinae*. Allergic reactions produce signs and symptoms that vary according to the site of the reaction, and sensitization may not be accompanied by the presence of any allergic symptoms (29). In addition, as HDMs are inhaled through the nasal leading to AR onset, it is anticipated that the severity of non-nasal symptoms are less likely to be influenced by HDMs sensitization.

It has been demonstrated that protease activation cascade (30) possibly functions differently in *D. farinae* and *D. pteronyssinus* due to the presence of different protease isoforms in the feces of these two species (31). The major contributor of allergenic potential of mites is found in their feces (32, 33). Different protease isoforms could induce different degree of reactions of the protein with protease-activated receptors (PARs) which ultimately influence allergenicity (34) as well as the cleavage of TJ molecules of epithelial cells (35–37).

In addition, the allergen found in *B. tropicalis*, Blo t 5, is structurally monomeric (38), while the allergen of *D. farinae*, Der f 5, is present as dimers (i.e., structure with large hydrophobic cavity) (39). The hydrophobic cavities in allergens are known to bind hydrophobic ligands, which are thought to stimulate the innate immune system and have adjuvant-like effects on IgE-mediated inflammatory responses in allergic diseases (38, 40). This suggests that Der f 5 confers greater immunogenicity than Blo t 5, and this at least partially explains why SPT wheal sizes of *D. farinae* sensitization demonstrated positive association with nasal and non-nasal symptoms severity in AR patients. However, the structure of *D. pteronyssinus* specific allergen is monomeric and it remains unknown why *D. pteronyssinus* sensitization, unlike *D. farinae* sensitization, is not associated with nasal and non-nasal symptoms severity scores.

Allergic inflammation results from not only cellular and humoral responses but also other mediators including neurosensory structures and growth factors such as neurotrophins (41, 42). Moreover, two biomarkers i.e., nasal nitric oxide and nasal cytology have been shown to predict treatment efficacy of sublingual immunotherapy in AR patients sensitized to HDMs (43). Future studies should also examine the potential contribution of these mediators and biomarkers to SPT wheal sizes and nasal or non-nasal manifestations in HDMs-sensitized AR patients.

We acknowledge the limitations of the study as follows: (1) Small sample size; (2) Sample size calculation had been conducted previously according to past gene expression studies, but not studies pertaining to SPT wheal sizes in AR patients, as this study was part of our previously published cross-sectional gene expression study (23); (3) Lack of correlation studies with other symptom questionnaires such as the total nasal symptom score (TNSS) (44–46), or with the levels of total and specific IgE. Future investigations addressing these limitations are thus recommended.

In conclusion, we demonstrated the significant correlation between wheal sizes of *D. farinae* sensitization with all nasal and majority of non-nasal symptoms severity scores. Such relationships were not observed in *D. pteronyssinus* and *B. tropicalis*. We suggest that increased SPT wheal sizes for *D. farinae* sensitization may predict the likelihood of more severe nasal severity in AR patients, and that these patients should be routinely monitored or managed to avoid the potential onset of severe nasal manifestations.

## Data Availability Statement

The original contributions presented in the study are included in the article's/Supplementary Material, further inquiries can be directed to the corresponding author.

## Ethics Statement

The studies involving human participants were reviewed and approved by Human Research Ethics Committee of Universiti Sains Malaysia (JEPeM) (approved code: USM/JEPeM/18060273). The patients/participants provided their written informed consent to participate in this study.

## Author Contributions

H-TT, NSMA, and KKW conceived the project and recruited research grants. SMNH, NSMA, and NMS recruited the participants and performed the investigations. SMNH prepared the tables and figures. KKW supervised data analysis. SMNH and KKW designed, wrote, and revised the manuscript.

## Funding

This work was supported by Universiti Sains Malaysia (USM) grants comprising of the Research University Grant (1001/PPSP/8012349) awarded to KKW and Research University Grant (1001.PPSP.8012285) awarded to NSMA.

## Conflict of Interest

The authors declare that the research was conducted in the absence of any commercial or financial relationships that could be construed as a potential conflict of interest.

## Publisher's Note

All claims expressed in this article are solely those of the authors and do not necessarily represent those of their affiliated organizations, or those of the publisher, the editors and the reviewers. Any product that may be evaluated in this article, or claim that may be made by its manufacturer, is not guaranteed or endorsed by the publisher.

## Supplementary Material

Supplementary Figure 1 Association of nasal symptoms severity scores with the number of HDM allergens sensitization of AR patients.

Supplementary Figure 2 Association of non-nasal symptoms severity scores with number of HDM allergens sensitization of AR patients.

Supplementary Table 1 The 7-point visual analog scale (VAS) indicator to assess AR nasal and non-nasal symptoms severity scores.
