# Supplementary material for: Higher Wheal Sizes of Dermatophagoides farinae Sensitization Exhibit Worse Nasal Symptoms in Allergic Rhinitis Patients
Source: Front Med (Lausanne). 2022 Feb 28;9:843432. doi: 10.3389/fmed.2022.843432 (PMC8918548; doi:10.3389/fmed.2022.843432)
Supplement: Supplementary Figure 1 — Association of nasal symptoms severity scores with the number of HDM allergens sensitization of AR patients. [file Data_Sheet_1.doc]

**Supplementary Figure 1.**


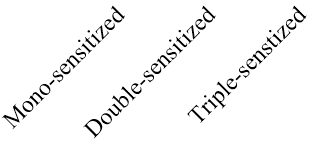

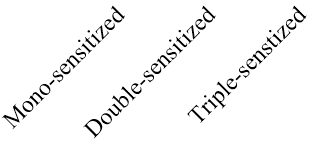

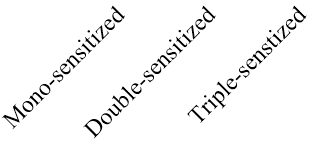


**Number of HDM sensitization**

**Number of HDM sensitization**

**Number of HDM sensitization**


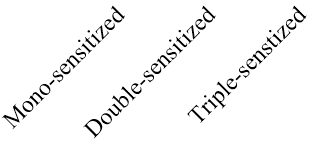

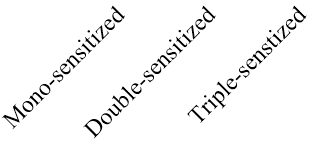


**Number of HDM sensitization**

**Number of HDM sensitization**

**Supplementary Figure 2**


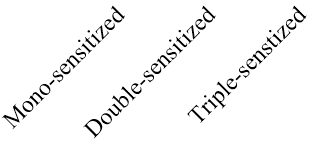

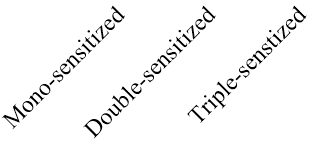

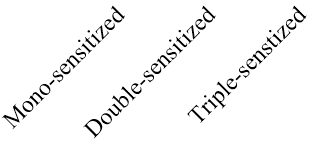


**Number of HDM sensitization**

**Number of HDM sensitization**

**Number of HDM sensitization**

**
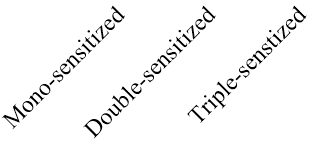

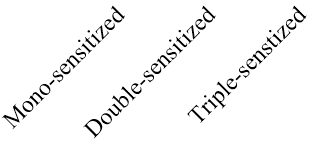

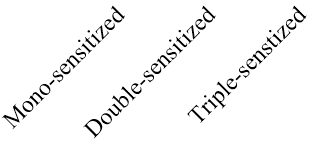
**

**Number of HDM sensitization**

**Number of HDM sensitization**

**Number of HDM sensitization**
