# Supplementary material for: Higher Wheal Sizes of Dermatophagoides farinae Sensitization Exhibit Worse Nasal Symptoms in Allergic Rhinitis Patients
Source: Front Med (Lausanne). 2022 Feb 28;9:843432. doi: 10.3389/fmed.2022.843432 (PMC8918548; doi:10.3389/fmed.2022.843432)
Supplement: Supplementary Table 1 — The 7-point visual analog scale (VAS) indicator to assess AR nasal and non-nasal symptoms severity scores. [file Table_1.DOCX]

**Supplementary Table 1.** The 7-point visual analogue scale (VAS) indicator to assess AR nasal and non-nasal symptoms severity scores.

| **Key to symptoms** |
| --- |
| 1 **None** – to an occasional limited episodes  2  3 **Mild** – steady symptoms but easily tolerable  4  5 **Moderately bothersome** – symptoms hard to tolerate, may interfere with activities of daily living and/or sleep  6  7 **Unbearable severe** – severe symptoms and unable to function all the time |
